# Transcriptome Analysis Identifies Novel Prognostic Genes in Osteosarcoma

**DOI:** 10.1155/2020/8081973

**Published:** 2020-10-06

**Authors:** Junfeng Chen, Xiaojun Guo, Guangjun Zeng, Jianhua Liu, Bin Zhao

**Affiliations:** ^1^Department of Orthopedics, Tianmen First People's Hospital, Tianmen, Hubei 431700, China; ^2^Department of Foot and Ankle Surgery, Wuhan Fourth Hospital, Tongji Medical College, Huazhong University of Science and Technology, Wuhan, Hubei, China

## Abstract

Osteosarcoma (OS), a malignant primary bone tumor often seen in young adults, is highly aggressive. The improvements in high-throughput technologies have accelerated the identification of various prognostic biomarkers for cancer survival prediction. However, only few studies focus on the prediction of prognosis in OS patients using gene expression data due to small sample size and the lack of public datasets. In the present study, the RNA-seq data of 82 OS samples, along with their clinical information, were collected from the TARGET database. To identify the prognostic genes for the OS survival prediction, we selected the top 50 genes of contribution as the initial candidate genes of the prognostic risk model, which were ranked by random forest model, and found that the prognostic model with five predictors including *CD180*, *MYC*, *PROSER2*, *DNAI1*, and *FATE1* was the optimal multivariable Cox regression model. Moreover, based on a multivariable Cox regression model, we also developed a scoring method and stratified the OS patients into groups of different risks. The stratification for OS patients in the validation set further demonstrated that our model has a robust performance. In addition, we also investigated the biological function of differentially expressed genes between two risk groups and found that those genes were mainly involved with biological pathways and processes regarding immunity. In summary, the identification of novel prognostic biomarkers in OS would greatly assist the prediction of OS survival and development of molecularly targeted therapies, which in turn benefit patients' survival.

## 1. Introduction

Osteosarcoma (OS), a malignant primary bone tumor often seen in young adults, is highly aggressive [1]. According to previous studies, OS patients without metastatic diseases present a survival of 70%, yet evidence suggests that metastases that take place at early stages result in worse prognosis [2]. OS can be further categorized into different groups as intramedullary and surface subtypes according to their histologic characteristics and is considered to be associated with multifactorial causes, and both genetic and environmental influences seem to have an impact on this disease [3]. However, for the majority of OS patients, its etiology still remains veiled. Patients' physique [4–6] and their genetic background [7], along with hormone secretion that could affect skeletal development [8], are all risk factors for OS. Currently, patients with OS mostly receive surgery and chemotherapy, which brings dramatic improvement in their long-term survival, yet accurate prognosis prediction is still required in making therapeutic decisions [9]. However, the only about 15-17% OS patients treated with only surgery could survive [10, 11]. In the early 1970s, the adjuvant chemotherapy was introduced and applied in the treatment of OS patients without metastatic disease [12]. Combined with surgery resection, current combinational chemotherapy could cure ~70% of OS patients. However, the five-year overall survival for patients with metastasis or relapse was still only about 20% [11, 13], voicing an urgent call for new therapies aimed at these patients.

With the advances in sequencing technologies, such as microarray, next-generation sequencing, and proteomics mass spectrum, the prognostic biomarkers for cancer survival prediction have been proposed by several studies [14–16]. Mutations in *TP53*, *RB1*, *CDKN2A*, *PTEN*, and *YAP1* [17] have been identified and widely observed using whole-genome sequencing (WGS) or whole-exome sequencing (WES) in OS patients, which greatly improved our understanding of the genomic landscape of OS. With next-generation sequencing, the identification of novel biomarkers becomes possible, which can not only broaden our insights into the pathogenic mechanism of OS but also provide the resource to build machine learning models to predict the prognosis of OS patients. For instance, *KRT5*, *HIPK2*, *MAP3K5*, and *CD5* were identified to serve as prognostic factors of osteosarcoma patients [18]. Moreover, risk predictive models based on one eight-gene [19] and one two-gene [20] (*PML*-*EPB41*) signatures have been built to predict overall survival of patients with osteosarcoma. However, only few studies focus on the prediction of prognosis in OS patients due to a small sample size using gene expression data and the lack of public datasets. In this study, we collected 82 OS samples with RNA-seq data and corresponding clinical data from the Therapeutically Applicable Research to Generate Effective Treatments (TARGET) database, selected prognostic genes, and built a prognostic risk model to assess and predict the overall survival of OS. The identification of novel prognostic biomarkers in OS would greatly assist the prediction of OS survival and the development of molecularly targeted therapies, which in turn benefit patients' survival.

## 2. Material and Methods

### 2.1. Data Sources

We obtained osteosarcoma RNA-seq data (TPM) and matched clinical data of OS patients from the TARGET database (https://ocg.cancer.gov/programs/target). A total of 82 patients from this dataset were constructed as a training set of our prognostic risk model. The dataset of GSE21257 [21] for further validation consisted of 34 osteosarcoma patients.

### 2.2. Screening of Genes for the Prognostic Risk Model of Osteosarcoma

First, a list of genes yielding TPM > 0.1 in more than half of the total samples was chosen for feature selection. Based on the clinical information and expression profiles of each patient, genes significantly associated with patients' survival were obtained by performing Cox regression analysis with the R Survival package. We further narrowed down this gene list based on the differential levels of prognostic outcomes, and genes whose *P* value is less than 0.01 were selected. Subsequently, these genes were ranked by the random forest algorithm in the R package randomForestSRC based on their relative contribution. Consequently, the top 50 genes were identified as the candidate genes to construct the risk model of osteosarcoma prognosis.

### 2.3. Model Construction and Evaluation

Utilizing the expression profiles of candidate prognostic genes and the survival data of patients, we built a prognosis risk model for OS using the multivariate Cox regression as previous studies described [22], and a list of genes that contributed significantly to this model was obtained, which consisted of our final candidates. We established a scoring formula for these finalized candidate genes to evaluate the risks for OS patients and used the median score to divide them into two subgroups, namely, high-risk and low-risk groups. Kaplan-Meier survival curves were plotted, respectively, for each group, and the differences in their survival were further assessed by the log-rank test.

### 2.4. Validation of the Prognostic Risk Model by an Independent Dataset

Validation dataset consisted of 34 osteosarcoma patients obtained from the GSE21257 dataset [21]. The prognostic risk scoring formula obtained from the training set was applied to evaluate the risk for each patient according to the expression of finalized candidate genes in each sample, accordingly. These patients were then labeled as those of high risk and of low risk based on the scores assigned to them, and their prognostic difference was further analyzed.

### 2.5. Gene Set Enrichment Analysis

Our prognostic risk model divided osteosarcoma patients into two categories, termed as high- and low-risk groups, and then differentially expressed genes (DEGs) between two groups were selected with two thresholds at ∣log2 (fold)  | >1 and *P* value < 0.05. Gene Ontology- (GO-) based enrichment analyses of these significantly differentially expressed genes were carried out in R with package clusterProfiler, as described in previous studies [23–25].

## 3. Results

### 3.1. Screening of Prognostic Genes for OS Survival Prediction

The gene expression data and corresponding clinical data of 82 patients were retrieved from the TARGET database. A total of 16,034 genes were introduced as variables in our prognostic risk model under the condition that these genes exhibited TPM > 0.1 in more than half of the total samples. Univariable Cox regression was performed, and Kaplan-Meier curves were plotted accordingly on all these genes, out of which 50 genes significantly related to the patient's survival were obtained (*P* values < 0.01). Subsequently, the contribution of these genes was ranked by the random forest algorithm, and the top 50 genes were selected as the initial candidate genes for the construction of the prognostic risk model.

**Figure 1:**
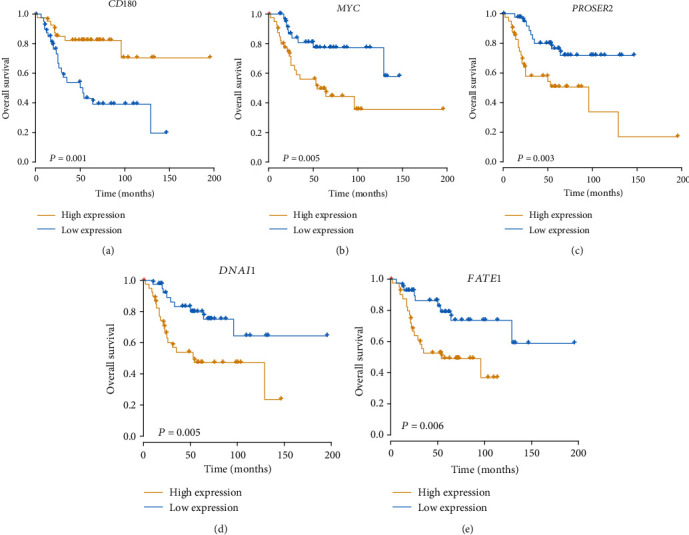
The Kaplan-Meier curves for samples stratified based on the expression levels of *CD180*, *MYC*, *PROSER2*, *DNAI1*, and *FATE1*, respectively. (a–e) Survival of OS patients stratified by expression of *CD180*, *MYC*, *PROSER2*, *DNAI1*, and *FATE1*, respectively, depicted by Kaplan-Meier plots.

Using those 50 genes, the prognostic risk model of osteosarcoma was developed, based on a multivariable cox regression. Among them, five genes, including *CD180*, *MYC*, *PROSER2*, *DNAI1*, and *FATE1*, with significant contribution to the model were selected as the candidate genes in the optimal prognostic risk model of osteosarcoma. Notably, the low expression of *CD180* and high expressions of *MYC*, *PROSER2*, *DNAI1*, and *FATE1* were identified to result in worse prognostic outcomes in OS (Figures 1(a)–1(e)). These results indicated that the five prognostic genes were highly associated with the OS prognosis.

### 3.2. Construction of Multivariable Cox Model Using Five Prognostic Genes

**Figure 2:**
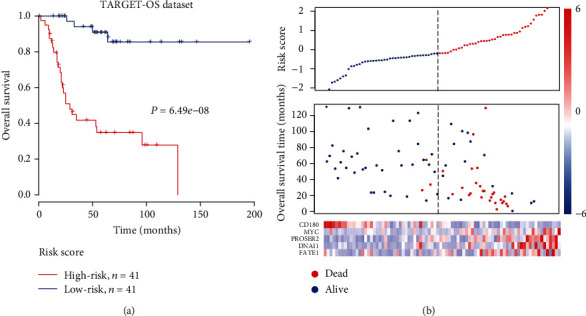
The risk stratification of the OS patients by the risk score in the training set (TARGET cohort): (a) Kaplan-Meier curves for patients in high-risk and low-risk groups in the training set stratified by risk scores; (b) the association of the risk scores with the survival time and status and expression levels of five genes. The samples were ranked by the risk scores.

Given the 5 genes, a multivariable Cox model was built to evaluate the risk of the OS patients. Specifically, the five genes showed significant association with the OS overall survival in both univariable and multivariable Cox models (Table 1). All 82 patients in the TARGET-osteosarcoma dataset were used as a training set, and we then estimated their risk scores according to the model. They were divided into high- and low-risk groups by the median risk score. Consistently, the overall survival of the high-risk group was significantly shorter than that of the low-risk group (Figure 2(a)). From this stratification, we noticed that the deceased patients of the high-risk group were found to be more than those in the low-risk group (Figure 2(b), *P* value < 0.05). In accordance with the Cox regression analyses, *CD180* was downregulated and another four genes were upregulated in the high-risk group (Figure 2(b)). These results indicated that the five genes acquired good fitting effect on the overall survival in OS.

### 3.3. Validation for the Prognostic Risk Model of OS

**Figure 3:**
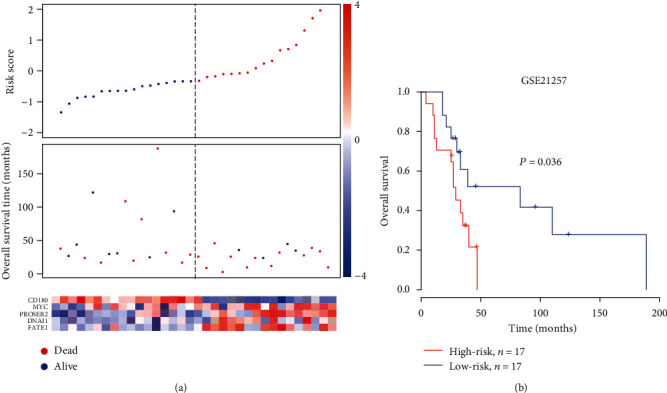
Prognostic risk model performance on the validation set (GSE21257 cohort). (a) The association of the risk scores with the survival time and status and expression levels of five genes in the validation set (GSE21257). The samples were ranked by the risk scores. (b) Kaplan-Meier curves for patients in the high-risk and low-risk groups in the validation set stratified by risk scores.

To validate the prognostic value of the five-gene-based Cox model, we collected an independent gene expression dataset with 34 OS samples from Gene Expression Omnibus (GEO) with accession GSE21257. The risk scores for 34 OS samples were estimated using the expression levels of the five genes in each individual. Similarly, these samples were also assigned into high-risk and low-risk groups by the median score. Consistently, in the high-risk group, we observed a greater number of deceased patients and shorter overall survival than the low-risk group (*P* value < 0.05, Figure 3(a)). The KM curves illustrated that patients of high risk exhibited significantly worse prognosis than those of the low-risk group (*P* value < 0.05, Figure 3(b)). Such significant difference in the overall survival time between two risk groups in the validation set suggested that the five-gene-signature Cox model could efficiently predict the prognostic risk in OS.

### 3.4. The Risk Score Based on the Five-Gene-Based Cox Model Serves as an Independent Prognostic Factor in OS

In order to evaluate the independence of this risk score, we conducted both univariable and multivariable Cox regression, using the risk score and three other clinical variables. In both univariable and multivariable Cox analyses, risk score was the only variable that correlated with the survival time (Table 2). Moreover, we found that white OS patients might have a lower risk than other ethnic groups in the multivariable Cox model with a lower statistical significance (*P* value < 0.1). These results suggested that risk score could function as an independent prognostic indicator in OS.

### 3.5. Biological Differences between the Two Risk Groups

**Figure 4:**
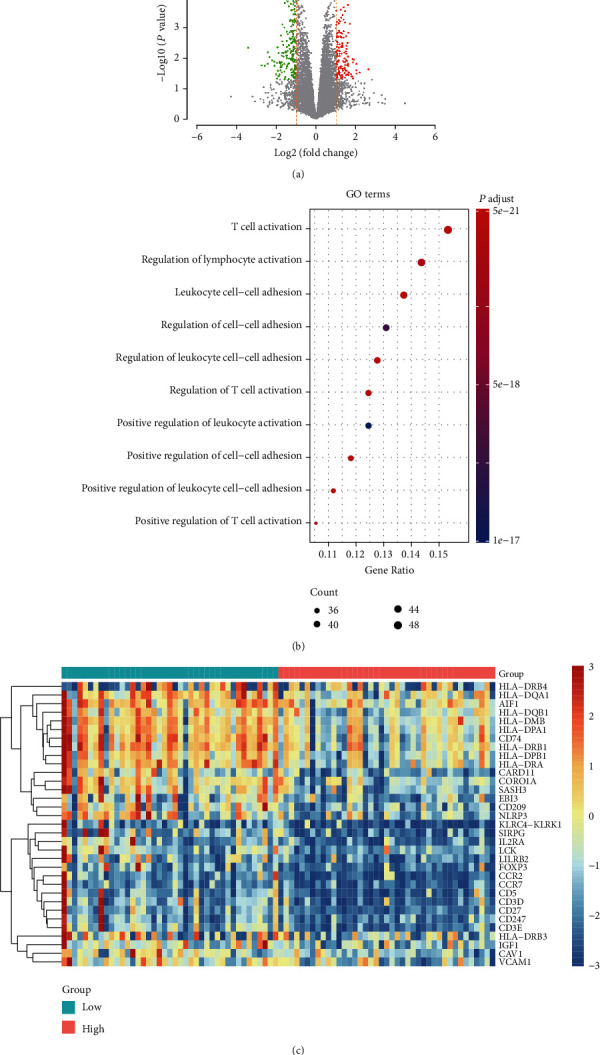
The differentially expressed genes (DEGs) and GO terms enriched by the DEGs between high- and low-risk groups. (a) The volcano plot displays the significantly up- and downregulated genes, which are colored by red and blue. (b) GO terms enriched by the differentially expressed genes. (c) The GO term-related genes downregulated in the high-risk group.

The differential expression analysis was performed for patients from the TARGET OS dataset, where patients were labeled as of high- and low-risk ones. A total of 351 significant differentially expressed genes were identified (thresholds: ∣log2 (fold change) | >1 and *P* value < 0.05), of which, compared with the low-risk group, 138 and 213 genes exhibited increased and decreased expression in the high-risk group (Figure 4(a)), respectively.

Gene Ontology- (GO-) based gene enrichment analysis revealed that immune-related GO terms, including leukocyte cell-cell adhesion and its T cell activation and its regulation, positive regulation of leukocyte cell-cell adhesion, and positive regulation of T cell activation, were highly enriched by these DEGs (Figure 4(b)), suggesting that the differed immune microenvironment of patients in high-risk and low-risk groups may be responsible for the difference in their prognostic outcomes. Further analysis revealed that major histocompatibility complex (MHC) class II genes were downregulated in the high-risk group (Figure 4(c)), suggesting that the lack of antigen processing and presentation might be associated with reduced immunity against tumor cells, thereby resulting in worse prognosis in OS. Collectively, the results suggested that the immune microenvironment of patients with osteosarcoma plays an essential role in OS patients' prognosis.

## 4. Discussion

The molecular basis behind OS tumorigenesis, progression, and metastasis has attracted growing attention. Though extensive researches have demonstrated the biological function of certain genes in OS, there is still a lack of effective biomarkers for OS survival prediction. Meanwhile, the prediction and comparison of the OS patients with different clinical outcomes could help clinicians make improvements in diagnostic or therapeutic strategies.

In the present study, 82 OS samples with RNA-seq data and matched clinical data were collected from the TARGET database. To identify the prognostic genes for the OS survival prediction, we selected the top 50 genes of contribution as the initial candidate genes of the prognostic risk model, which were ranked by the random forest model. Multivariable Cox regression analysis suggested that the prognostic model with five predictors including CD180, MYC, PROSER2, DNAI1, and FATE1 was the optimal multivariable Cox regression model. Among the five prognostic genes, only *CD180*, which could lead to NF-kappa-B activation [26], was negatively correlated with the OS survival. As we know, CD180 is a cell surface molecule of lymphocytes, and its high expression may indicate the high anticancer activity of lymphocytes, thereby suppressing the growth of OS tumors. CD180, as well as CCR2, has been identified as robust pharmacodynamic tumor and blood biomarkers for clinical use with BRD4/BET inhibitors [27]. In addition to *DNAI1*, another three genes, *MYC* [28], *PROSER2* [29], and *FATE1* [30], have been reported to be associated with several cancers.

Moreover, we also stratified the OS patients into high-risk and low-risk groups according to the risk score estimated by the multivariable Cox regression model. The stratification for OS patients in the validation set based on risk scores predicted by the multivariable Cox regression model further demonstrated that our model was significant and robust (log-rank test, *P* < 0.05). It should be noted that only 34 primary tissues were used in the validation set, which might be a major limitation for the five-gene-based predictive model. In addition, we also investigated the biological differences between the high-risk and low-risk groups and found that biological processes regarding immunity were highly enriched by the differentially expressed genes between the two risk groups. The expression of MHC II class genes was reduced in high-risk OS samples (Figure 4(c)), suggesting that these samples might lose the abilities of presenting and processing extracellular pathogens. Consistently, MHC class II genes were downregulated and associated with unfavorable outcome in OS [31, 32].

In summary, we established a prognostic risk model of five genes in osteosarcoma, stratified the osteosarcoma samples into high-risk and low-risk groups, and uncovered the underlying molecular mechanism associated with the prognosis, which not only provided some evidence for related researchers but also improved our understanding of OS prognosis.

## Figures and Tables

**Table 1:** The summary of prognostic values for the five genes in univariable and multivariable Cox regression models.

Features	Univariable cox regression		Multivariable cox regression	
	Hazard ratio	*P* value	Hazard ratio	*P* value
*CD180*	0.43	2.19*E*-03	0.44	4.98*E*-03
*MYC*	1.01	9.60*E*-05	1.01	1.18*E*-05
*PROSER2*	1.10	6.52*E*-06	1.09	4.60*E*-03
*DNAI1*	1.53	8.54*E*-06	1.42	2.19*E*-03
*FATE1*	6.08	2.62*E*-05	7.05	4.05*E*-06

**Table 2:** The statistical significance of the risk score in univariable and in multivariable Cox regression models with other clinical parameters.

Features	Univariable cox regression				Multivariable cox regression			
	*P* value	HR	Lower 95% CI	Upper 95% CI	*P* value	HR	Lower 95% CI	Upper 95% CI
Risk score	4.42*E*-12	19.7	4.64	15.6	4.34*E*-11	12.3	5.85	26.1
Gender (female/male)	0.30	0.68	0.33	1.41	0.17	0.54	0.23	1.29
Race (white/other)	0.23	0.64	0.30	1.34	0.07	0.47	0.21	1.08
Age	0.82	1	1	1	0.82	1	1	1

## Data Availability

Osteosarcoma RNA-seq data (TPM) and matched clinical data of OS patients from the TARGET database (https://ocg.cancer.gov/programs/target) and the dataset of GSE21257 were used for further validation.
